# Analysis of p-Si macropore etching using FFT-impedance spectroscopy

**DOI:** 10.1186/1556-276X-7-320

**Published:** 2012-06-20

**Authors:** Emmanuel Ossei-Wusu, Jürgen Carstensen, Helmut Föll

**Affiliations:** 1 Institute for Materials Science, Christian-Albrechts-University of Kiel, Kaiserstrasse 2, Kiel, 24143, Germany

**Keywords:** p-Si macropores, HF concentration, water content or concentration, organic electrolytes, impedance spectroscopy

## Abstract

The dependence of the etch mechanism of lithographically seeded macropores in low-doped p-type silicon on water and hydrofluoric acid (HF) concentrations has been investigated. Using different HF concentrations (prepared from 48 and 73 wt.% HF) in organic electrolytes, the pore morphologies of etched samples have been related to *in situ* impedance spectra (IS) obtained by Fast Fourier Transform (FFT) technique. It will be shown that most of the data can be fitted with a simple equivalent circuit model. The model predicts that the HF concentration is responsible for the net silicon dissolution rate, while the dissolution rate selectivity at the pore tips and walls that ultimately enables pore etching depends on the water content. The ‘quality’ of the pores increases with decreasing water content in HF/organic electrolytes.

## Background

The formation of macropores in silicon was first discovered by Lehmann and Föll in the 1990s [1]; macropores in this case were etched into n-type silicon under backside illumination using HF/aqueous electrolytes. They came up with a model (usually referred to as the Lehmann model) that essentially relied on the geometry of the space charge region to explain pore formation. The model is only applicable to n-type silicon, and it took almost half a decade before Propst and Kohl succeeded for the first time in etching macropores in p-type silicon (with rather ‘bad’ pore morphologies) using acetonitrile as a base for an almost water-free electrolyte [2]. Since then, the use of electrolytes based on organic solvents such as dimethylformamide (DMF), dimethylacetamide (DMA), dimethyl sulfoxide, and ethanol have been shown to enable macropore etching in p-type silicon, and several models emerged dealing with this. For example, Ponomarev and Lévy-Clément [3,4] attempted to correlate the etching of macropores in p-type Si to a covering (and thus passivation) of the pore walls by the organic molecules, whereas Wehrspohn et al. emphasized differential current flow based on the resistivity of the wafer and the electrolytes and even reported the first p-macropores etched by using aqueous electrolytes [5]. In 2000, Christophersen et al. proposed H-termination as the passivation mechanism of the pore walls [6]. Meanwhile, it was shown that lithographically pre-structuring the sample has a major influence on the ease of macropore etching in p-type substrates and the quality of the pores (far more pronounced than in n-type substrates) [7-10]. Not much data seem to exist about the role of the HF concentration in p-macropore formation.

In this investigation, emphasis was placed on the effect of the HF and water concentrations while using organic solvents as major ingredient of the electrolytes. The different morphologies of the pores etched, as well as the importance on the dissociation rate of HF in the solvents, will be reported. FFT impedance spectra, which were taken *in situ* every 2 s, were analyzed and compared to the final pore morphologies.

## Methods

The starting material for this investigation was mono-crystalline, boron-doped p-type silicon wafers with {100} orientation, a resistivity of nominally 20 Ωcm (doping concentration 10^14^ cm^−3^), and a standard backside aluminum metallization for good ohmic contacts. In order to ensure uniform pore growth from the very beginning [8], lithographically pre-structured p-Si wafers were used. Structuring of the wafers was done by our group (using the standard technology), resulting in a cubic arrangement of inverted pyramids with a spacing of 2 to 3 μm. Details of the structuring procedure are described elsewhere [11]. The wafers were cut into 2 × 2 cm^2^ pieces for the pore etching, with an effective etching area of 1 cm^2^. Etching was performed at a constant temperature of 20 °C. Electrolytes were based on either DMF or DMA. Hydrofluoric acid was added to produce four different concentrations: 2.5, 5, 7.5, and 10 wt.%, respectively. Two kinds of HF were used: 48 and 73 wt.%. This results in eight kinds of electrolyte that differ not only in their HF concentration but also in the residual nominal water concentration. Table 1 lists the resulting properties. In what follows, we use a shorthand description for electrolytes by using a string that lists solvent/HF concentration/(water concentration). Note that the water concentration in a DMF 5%(5.4%) electrolyte is about the same as in a DMF 10%(3.8%) electrolyte and that this allows evaluation of the influence of the HF concentration independently from the influence of the water concentration.

**Table 1:** HF and water concentrations in the various electrolytes used

	**2.5 wt.% HF**		**5 wt.% HF**		**7.5 wt.% HF**		**10 wt.% HF**	
HF (wt.%)	48	73	48	73	48	73	48	73
H_2_O (wt.%)	2.7	0.7	5.4	1.5	8.1	2.5	11	3.8

Concentrations are always given in weight percent so that differences in the densities of the organic electrolytes were not relevant. The experiments with DMA 2.5%(x) electrolytes always produced rather ‘bad’ pore morphologies and were therefore ignored.

While the use of these organic solvents is not new in the electrochemistry of silicon [2,3,8,12,13], in this work, we used only newly prepared electrolyte to perform the experiments, and the volume of the organic solvents used for each preparation was the same (190 ml) for all the experiments, ensuring comparable residual contents of water. The pores were etched for 30 min with a constant current density of *j =* 10 mA/cm^2^. *In situ* FFT-IS measurements were performed every 2 s; recording a spectrum took 1 s. The IS employed 24 frequencies in a range of 5 Hz to 20 kHz. All spectra could be fitted quite well by a simple equivalent circuit consisting of one series resistance in series with three circuits with a resistor parallel to a capacitor (*R*_p_*C*_p_ components). Thus, there are seven independent *R*/*C* parameters, and these parameters were extracted *in situ*. From these parameters, three time-constant *τ* = *RC* that were also displayed *in situ* could be constructed. To the best of our knowledge, this is thus the first investigation of p-macropores where relevant data other than currents and potentials were obtained during etching. A detailed description of the model and procedures with respect to *in situ* FFT impedance are described in [14-16]. A fully integrated laboratory etching system of ET&TE GmbH (Kiel, Germany) controlled all parameters of the etching procedure. The pore morphologies were examined by a scanning electronic microscope and were compared with the FFT-IS measurements for further analysis.

## Results

### Pore morphology and geometry

**Figure 1:**
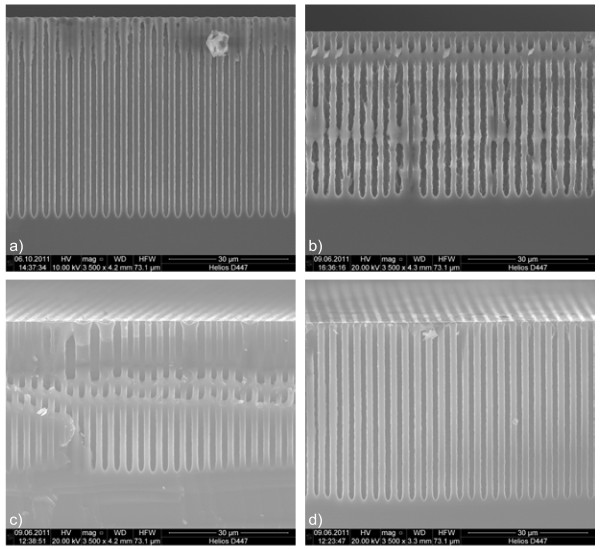
** Macropore morphologies obtained with DMA- and DMF-based electrolytes and different HF and water concentrations.** The pores were etched into 20-Ω cm p-Si {100} with nuclei distance of 3 μm and *j* = 10 mA cm^−2^. **a**) DMF 5%(5.4%) resulted in pores with smooth walls and constant diameter. **b**) DMF 10%(11%) resulted in pores with rougher walls, larger diameter, and shorter lengths. **c**) DMA 10%(11%) resulted in pores with smooth walls, constant but bigger diameter, and shorter lengths. **d**) DMA 10%(3.8%) resulted in pores with geometries nearly identical to that of (a).

The parameters for etching the ‘best’ macropores are used as reference. The best pores are pores with straight and smooth walls, having a constant cross-section, and growing deep and uniform into the substrate. The best pores were obtained with a DMF 5%(5.4%) electrolyte at a temperature of 20 °C and a current density of 10 mA/cm^2^ applied for 30 min. These best pores have smooth walls, a constant diameter (around 1.4 μm), and pore lengths of around 50 μm (Figure 1a). Against expectations, experiments using the same parameters except for an increased HF concentration, i.e., DMF 10%(11%) instead of DMF 5%(5.4%), produced pores with shorter lengths (around 42 μm), rougher walls, and larger diameters, as shown in Figure 1b.

On the other hand, maintaining the HF/water concentration at 10%(11%) and repeating the experiment using DMA as a solvent, the etched pores had much smoother pore walls (Figure 1c) similar to those etched with DMF 5%(5.4%) (Figure 1a) but were shorter in length (around 40 μm) and slightly bigger in diameter (around 1.7 μm). Further, the morphology of the pores etched using DMA 10%(3.8%) shown in Figure 1d was nearly identical to that of the pores etched with DMF 5%(5.4%) in Figure 1a. This shows that both the HF concentration and the water content are important for the pore formation. For almost the same water content, DMA needs approximately double the HF concentration in comparison to DMF to produce the same pore structure. We postulate that this is linked to the different dissociation rates of the HF in the solvents, which is well known to have a big impact on the pore etching [13,17]. It should be mentioned that pores etched with DMA 5%(5.4%) electrolyte had smooth walls but shorter lengths (around 20 μm) and bigger diameters (around 2.2 μm) than those produced with DMA 10%(3.8%).

**Figure 2:**
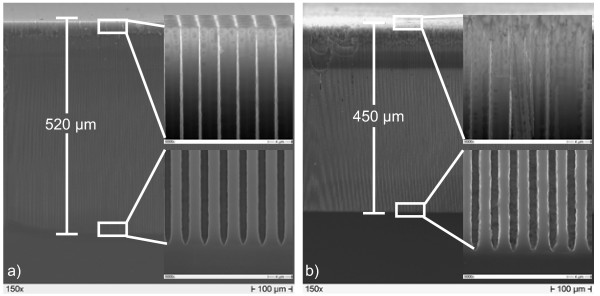
** Extremely deep ‘good’ pores etched with different water concentrations in DMF.a**) DMF 5%(1.5%) and **b**) DMF 5%(5.4%).

To double-check the effect of water content on the pore lengths, two experiments were designed for producing very deep pores. DMF 5%(5.4%) and DMF 5%(1.5%) electrolytes were employed for this. The etching time was 540 min, and an optimized current density profile was used. Etching started with 3 mA/cm^2^ for 2 min, followed by a sharp increase (within 1 min) to 23 mA/cm^2^, a linear decrease to 10 mA/cm^2^ within 120 min, and finally another linear decrease to 5.7 mA/cm^2^ within 417 min. As expected, pores etched with DMF 5%(1.5%) with less water concentration were found to be around 520 μm deep and, thus, 70 μm deeper and smoother than those etched with the DMF 5%(5.4%) where the water content was relatively high. The pores obtained are shown in Figure 2.

### FFT-IS results

**Figure 3:**
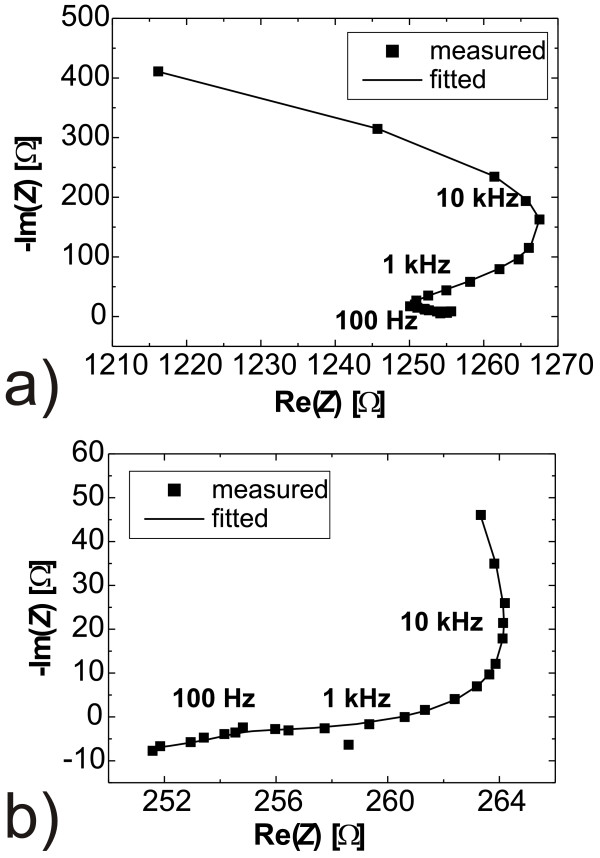
** Nyquist plots showing the high-quality fitting (solid line) for substantially different HF and water concentrations.** Pores were etched into 20 Ωcm p-Si {100) at *j* = 10 mAcm^2^. **a**) DMF 5%(5.4%) and **b**) DMF 10%(11%).

For all experiments, FFT-IS has been performed every 2 s. Many-thousand spectra could be well fitted to a model containing three (*R*_p_*C*_p_) components and one *R*_s_ in series; the Nyquist plots in Figure 3 show examples. These plots resulted during pore etching with DMF 5%(5.4%) and DMF 10%(11%) electrolytes. Those pores do not only differ in quality, as outlined above, but also in the quantitative impedance data. Not only the shape of the curves is rather different, but the impedance values also differ by a factor of 10 and 5, respectively, on the imaginary and real axes. Nevertheless, the data can be fitted rather well with one model, as outlined above.

**Figure 4:**
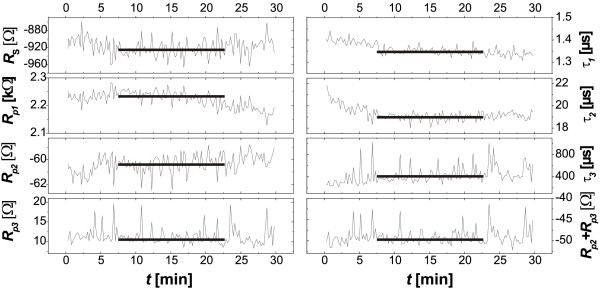
** Example of*in situ*extractions of fitting parameters and additional calculated parameters.** The data show the stability of the pore etching process after a nucleation period of around 7 min. The short horizontal lines show the average of the steady state values that are used in what follows. Pores here were etched with DMF 5%(5.4%).

The model fits the overwhelming majority of the remaining many-thousand impedance spectra just as well. Note that it was not possible to use models with less than three (*R*_p_*C*_p_) components to fit all pore-etching experimental parameters. The spectra thus generated seven individual parameters plus several calculated ones, e.g., time constants or (*R*_p2_ + *R*_p3_) as a function of time. These parameters were extracted *in situ* and displayed during the etching. Figure 4 shows a typical example with data generated during pore etching with DMF 5%(5.4%).

**Figure 5:**
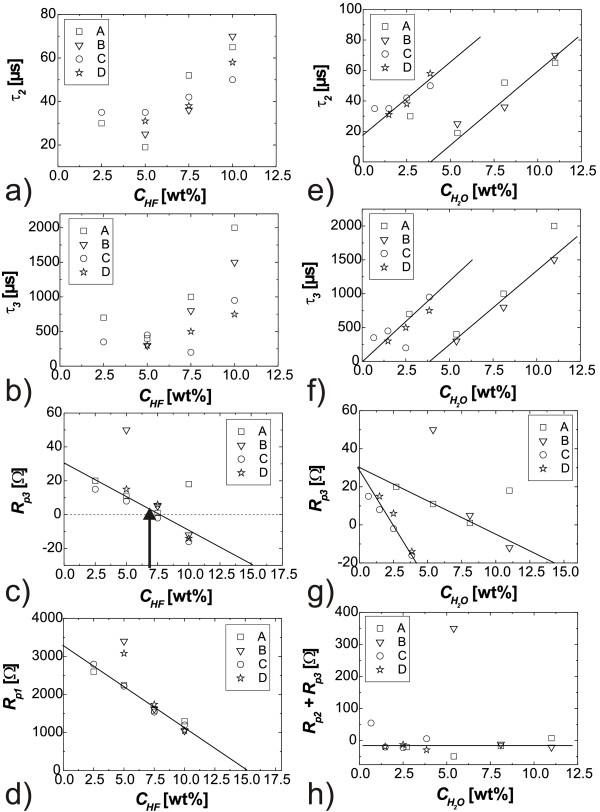
** The effect of HF and/or water concentrations on six fitting parameters (extracted from Figure 4).** A, 48 wt.% HF/DMF; B, 48 wt.% HF/DMA; C, 73 wt.% HF/DMF; and D, 73 wt.% HF/DMA electrolytes. **a**) and **b**) show no clear dependence of the time constants (*τ*_2_ and *τ*_3_) on the HF concentration; **c**) and **d**) show that the HF concentration is proportional to the transient resistances (*R*_p1_ and *R*_p3_); **e**) and **f**) show the effect of water on the time constants (*τ*_2_ and *τ*_3_); **g**) shows no clear effect of the water concentration on *R*_p3_; and **h**) shows that neither the HF concentration nor the water content has effect on the sum of the transient resistances (*R*_p2_ and *R*_p3_).

The individual parameter curves generated stay rather constant during the bulk of etching (the ‘noise’ visible in Figure 4 is mostly due to the expanded scales and relatively small in absolute values), which demonstrates the stability of the etching process during the complete etching time of 30 min. From these curves, the steady state values (indicated by straight horizontal lines) of all fitting parameters have been extracted and plotted versus HF and/or water concentrations, as shown in Figure 5.

## Discussions

Pore etching is a very complicated process in which all the parameters involved should be in their optimal regime to produce comparable results. Pore formation mechanisms, which have been studied in detail by many authors [1,2,18-20], take into consideration the nucleation of the pores, oxide formation and dissolution, diffusion limitations and thus local changes of, e.g., the HF concentration as the pores get longer, current flow at the pore tips related to local field strengths, the leakage current (current that flows in the dark) through the pore walls, the selectivity of the hydrogen (H_2_) passivation of the pore walls and tips, and much more.

Detailed descriptions of the importance of doping concentration, lithography pre-structuring KOH etch pits, and the kind of solvent can be found in [8,9,21-25]. None of the models, however, has anything to say on the impedance underlying the etching process. Here, we will limit the discussion to the data presented, except to point out that, without the seeded nucleation, no good pores would have been obtained in most cases and that most of the models mentioned above do not consider the influence of nucleation on pores.

DMF 5%(5.4%) and DMA 10%(3.8%) produced nearly identical pores as far as morphologies are concerned. This most likely indicates that the dissociation rate of HF in a particular solvent is an (obviously) important parameter, which has impact on the pore formation (as it has also been mentioned by [13,17]). If, for the time being, one accepts this as the reason for the difference between the solvents, it follows that the HF dissociation in DMA should be smaller than in DMF.

In this paper, we will only address some more general features, which can be extracted from the FFT impedance data shown in Figure 5. Most astonishing is the result shown in Figure 5h; the sum of the transfer resistances *R*_p2_ and *R*_p3_ is constant and thus independent of the water concentration (and of the HF concentration). Although the reason for this is not clear, it cannot be treated as a trivial or self-explaining result. Like in the case of energy conservation, system parameters that do not change but are conserved hint at fundamental properties of a system. Here, the sum of the two transfer resistances that are related to the large time constants *τ*_2_ and *τ*_3_ (i.e., the slow processes) stays constant. To some extent, this can be seen as a necessity for etching ‘good’ macropores in p-Si since constancy of *R*_p2_ + *R*_p3_ is not a general feature of macropore formation. Many experiments that produce only ‘bad’ pores do not show this effect. This indicates strongly that the slow processes related to the large time constants are essential for the formation of good macropores in p-Si. Comparing Figure 5a and Figure 5e or Figure 5b and Figure 5f, respectively, the two large time constants show a simple dependence on the water concentration (two straight lines), while the relation to the HF concentration is not that clear. Thus, it is probably the water concentration which has the most direct impact on the two slow processes.

Comparing Figure 5c and Figure 5g, the transfer resistance *R*_p3_ depends linearly on the HF concentration, while two straight lines are found as a function of water concentration, so in contrast to the time constants, the transfer resistances depend mainly on the HF concentration. The sum of the three transfer resistances just describes the linear response in the steady state (i.e., *ω* ≥ 0); in other words, the amount of silicon etched away depends, as clearly expected, mainly on the HF concentration. In contrast, the pore quality depends decisively on the water concentration. High pore quality is achieved if etching proceeds only at pore tips and not at the pore walls; the results thus give strong indications that, for p-Si, the water concentration in the organic solvent has a strong impact on the passivation of the pore walls. The results shown in Figure 2 may be seen as a direct check of this statement. Although the electrolyte DMF 5%(5.4%) allows for very good pore formation when etching for 30 min, reaching a pore depth of 50 μm, strong differences in using DMF 5%(1.5%) are found when etching for much longer times (540 min), reaching pore depths of 450 and 520 μm, respectively. The insets show the large differences especially in the morphology of the pore walls. In the experiment using higher water concentration, the walls are rougher close to the pore tips, and close to the top, the pore walls are nearly destroyed, while for DMF 5%(1.5%), the pore walls are much more stable and smoother.

## Conclusions

It has been demonstrated that the HF concentration and the water content of DMA- and DMF-based electrolytes are essential for the quality of macropores etched into lithographically pre-structured low-doped p-type silicon. The achievable pore lengths, pore diameters, and the pore wall smoothness depend on both parameters (and the solvent). From *in situ* impedance data, it can be concluded that the HF concentration is mainly responsible for the volume of silicon dissolved, while the water content is instrumental for the selectivity of the etching between pore tips and walls. The water content is thus instrumental for the pore wall stability and, therefore, the maximum achievable lengths of the pores.

## Competing interests

The authors declare that they have no competing interests.

## Authors’ contributions

All authors have contributed equally to this work, read, and approved the final manuscript.
